# Acquired Immunity Is Not Essential for Radiation-Induced Heart Dysfunction but Exerts a Complex Impact on Injury

**DOI:** 10.3390/cancers12040983

**Published:** 2020-04-16

**Authors:** Rachel A. Schlaak, Anne Frei, Brian L. Fish, Leanne Harmann, Tracy Gasperetti, Jamie L. Pipke, Yunguang Sun, Hallgeir Rui, Michael J. Flister, Benjamin N. Gantner, Carmen Bergom

**Affiliations:** 1 Department of Pharmacology & Toxicology, Medical College of Wisconsin, Milwaukee, WI 53226, USA; rachelmeyer@mcw.edu; 2 Department of Radiation Oncology, Medical College of Wisconsin, Milwaukee, WI 53226, USA; afrei@mcw.edu (A.F.); bfish@mcw.edu (B.L.F.); tgasperetti@mcw.edu (T.G.); jpipke@mcw.edu (J.L.P.); 3 Department of Medicine, Division of Cardiovascular Medicine, Medical College of Wisconsin, Milwaukee WI 53226, USA; lharmann@mcw.edu; 4 Department of Pathology, Medical College of Wisconsin, Milwaukee, WI 53226, USA; ysun@mcw.edu (Y.S.); hrui@mcw.edu (H.R.); 5 Cancer Center, Medical College of Wisconsin, Milwaukee, WI 53226, USA; mflister@mcw.edu (M.J.F.); bgantner@mcw.edu (B.N.G.); 6 Cardiovascular Center, Medical College of Wisconsin, Milwaukee, WI 53226, USA; 7 Department of Physiology, Medical College of Wisconsin, Milwaukee, WI 53226, USA; 8 Genomic Sciences and Precision Medicine Center, Medical College of Wisconsin, Milwaukee, WI 53226, USA; 9 Department of Medicine, Division of Endocrinology, Medical College of Wisconsin, Milwaukee, WI 53226, USA

**Keywords:** radiation-induced heart disease, cardiac radiation, cardiotoxicity, immune-compromised, IL-2 receptor gamma knockout, lymphocytes, T cells, radiation, immuno-oncology

## Abstract

While radiation therapy (RT) can improve cancer outcomes, it can lead to radiation-induced heart dysfunction (RIHD) in patients with thoracic tumors. This study examines the role of adaptive immune cells in RIHD. In Salt-Sensitive (SS) rats, image-guided whole-heart RT increased cardiac T-cell infiltration. We analyzed the functional requirement for these cells in RIHD using a genetic model of T- and B-cell deficiency (interleukin-2 receptor gamma chain knockout (IL2RG^−/−^)) and observed a complex role for these cells. Surprisingly, while IL2RG deficiency conferred protection from cardiac hypertrophy, it worsened heart function via echocardiogram three months after a large single RT dose, including increased end-systolic volume (ESV) and reduced ejection fraction (EF) and fractional shortening (FS) (*p* < 0.05). Fractionated RT, however, did not yield similarly increased injury. Our results indicate that T cells are not uniformly required for RIHD in this model, nor do they account for our previously reported differences in cardiac RT sensitivity between SS and SS.BN3 rats. The increasing use of immunotherapies in conjunction with traditional cancer treatments demands better models to study the interactions between immunity and RT for effective therapy. We present a model that reveals complex roles for adaptive immune cells in cardiac injury that vary depending on clinically relevant factors, including RT dose/fractionation, sex, and genetic background.

## 1. Introduction

Radiation therapy (RT) is established as a major modality in treating malignancies, with over 50% of cancer patients receiving RT. While RT is used to treat the tumor, toxic side effects to surrounding normal tissues can occur, and normal tissue radiation exposure increases the potential for acute and/or chronic complications in cancer patients. As cancer therapies continue to improve, the number of cancer survivors continues to grow, adding to the global cancer burden. This concern is amplified in thoracic cancers where protecting the heart is a major concern. RT exposure is a recognized risk factor for cardiovascular morbidity and death [1,2,3]. The most common RT-induced heart and vascular toxicities, collectively referred to as radiation-induced heart dysfunction (RIHD), include pericarditis, ischemic heart disease, conduction abnormalities, myocardial fibrosis, and dysfunctional valves [2,3,4,5,6]. RIHD presents months to years after RT and contributes significantly to increased rates of morbidity and mortality [7,8]. Current radiation treatment regimens often use techniques that reduce cardiac radiation exposure and associated risks [9,10,11,12,13]. Recent evidence, however, shows that cardiac morbidity remains high for patients with thoracic cancers, including Hodgkin lymphoma [14], breast cancer [15], and lung cancer [16]. Therefore, a better understanding of normal heart tissue responses to radiation injury is necessary in order to determine what mechanisms lead to radiation-induced cardiovascular disease. Targeted interventions could be developed that prevent and/or treat RIHD while maintaining radiation doses needed for maximum tumor control.

RT aims to target rapidly proliferating cancer cells, but also affects normal cells at the site of treatment. Thoracic radiation therapy can affect the heart, damaging cardiomyocytes, fibroblasts, and/or endothelial cells [17]. Damage to these and other cell populations can release “danger signals” that trigger inflammatory processes and activate immune cells [18,19]. Inflammation, however, can also contribute to heart homeostasis by promoting tissue remodeling and removing damaged tissue from sites of injury [20,21]. Further complicating this picture, RT can directly affect immune cells, with the effects of cardiac radiation varying dramatically between cell types. Sensitivity among lymphocytes ranges from radiosensitive B cells, naïve T helper (Th) cells [22], and natural killer (NK) cells, to more radioresistant cells like T memory cells [23], NK T cells [24], and regulatory T cells [25]. In general, this correlates with differences in proliferation since non-proliferating cells tend to be more radioresistant and demonstrate decreased rates of apoptosis [19]. Despite a basic knowledge of radiation can alter immunity, there is a need for better physiologic models to study how immune dysfunction contributes to RIHD.

Due to the increasing use of immune checkpoint inhibitors in cancer patients, as well as the variable state of adaptive immune cells in patients receiving chemotherapy and RT, it is important to better understand how adaptive immune cells alter the development and severity of RIHD. Using our previously published rat model of RIHD [26], we examined the role of T cells in cardiac radiation injury. We found that T-cell infiltration occurs in the heart after RT, and that localized heart irradiation leads to secretion of the T-cell-associated cytokines interleukin 2 (IL-2) and IL-13 [27,28]. This led us to examine the contribution of a mature adaptive immune system using a well-accepted genetic strategy to delete acquired immune cells to determine their role in RIHD. Mouse studies used *IL2rg* gene disruption to create a model of x-linked severe combined immunodeficiency (X-SCID), where the immunodeficient phenotypes are characterized by near complete lack of T cells, B cells, and NK cells [29,30,31]. Mashimo and colleagues generated interleukin-2 receptor gamma chain knockout (IL2RG^−/−^) rats in the F344/Stm background using zinc-finger nucleases and reported that IL2RG^−/−^ rats display similar immune deficiencies as the mouse model [32]. This zinc-finger nuclease approach was also performed on inbred Salt-Sensitive (SS) rats to create IL2RG^−/−^ rats [33,34]. The immune-competent SS rat strain previously demonstrated increased sensitivity to localized cardiac RT compared to the Brown Norway (BN) strain [26]. Here, we report that SS IL2RG^−/−^ rats also developed RIHD after localized high dose irradiation, with worse heart function measured via echocardiogram at three months post-RT compared to the SS immunocompetent (SS WT) rats. Additionally, the SS IL2RG^−/−^ rats did not exhibit hypertrophy after RT compared to sham treatment, although the SS WT female exhibited cardiac hypertrophy at five months post-RT. The changes in RIHD severity in the IL2RG^−/−^ rats, compared to results from WT immunocompetent rats, were overall revealed to be complex, with differing results dependent on treatment regimen (dose and fractionation), sex, and genetic background. We demonstrate that, while T cells are not essential for cardiac injury in response to RT, acquired immune cells can contribute in complex ways to the severity of injury in a manner that is dependent on clinically relevant factors. These findings highlight the importance of a more detailed understanding of how immune cells shape the response to cardiac radiation exposure in light of the increased prevalence of immune checkpoint inhibitors and other immunotherapies in cancer treatment.

## 2. Results

### 2.1. Circulating IL-2 and IL-13 Levels Increase in SS Rats Following Localized Cardiac Radiation

**Figure 1:**
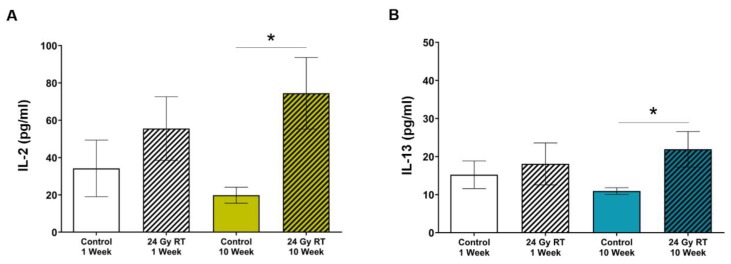
Salt-Sensitive (SS) wild-type (WT) rats have increased concentrations of circulating interleukin 2 (IL-2) and IL-13 after localized cardiac radiation. Adult SS WT female rats were administered either 24 Gy of localized cardiac radiation in one fraction or sham radiation. (**A**) A statistically significant increased concentration of IL-2 was detected in plasma 10 weeks post-radiation therapy (RT) compared to sham-treated. (**B**) Statistically significant increases in IL-13 were detected in plasma at 10 weeks post-RT compared to plasma from age-matched sham-treated rats. Plasma was collected at the time of harvest at one or 10 weeks post-RT (*n* = 7–10/group, * *p* < 0.05).

We previously reported that SS immune-competent female rats administered one fraction of 24 Gy develop cardiac hypertrophy, systolic dysfunction, and pleural and pericardial effusions at three and five months after RT compared to control rats [26]. We investigated the consequences of RT treatment on inflammatory cytokine responses and immune cell recruitment with the goal of understanding how they might contribute to RIHD. Plasma was isolated from female SS rats at one and 10 weeks post-RT and analyzed using cytokine arrays. We chose the 10-week time point to explore potential inflammatory mechanisms underlying the signs of left-sided heart failure that are seen on echocardiograms at 12 weeks post-RT [26]. SS rats showed a trend toward increased circulating IL-2 one week after treatment, with significantly elevated levels at 10 weeks, when compared to age-matched sham-treated animals (Figure 1A, *p* = 0.02). There were increased levels of IL-13 at 10 weeks post-RT as well (Figure 1B, *p* = 0.03). These results suggested a possible role for T cells in RIHD resulting from cardiac RT in the SS rats [28,35,36,37,38,39,40]. We then examined whether the T cell compartment may play a role in RIHD after cardiac RT in the SS rats by examining infiltrating T cells. 

### 2.2. Cluster of Differentiation 3-Positive (CD3^+^) and CD8α^+^ T-Cell Populations Increase in Heart Tissue at 10 Weeks Post-RT

**Figure 2:**
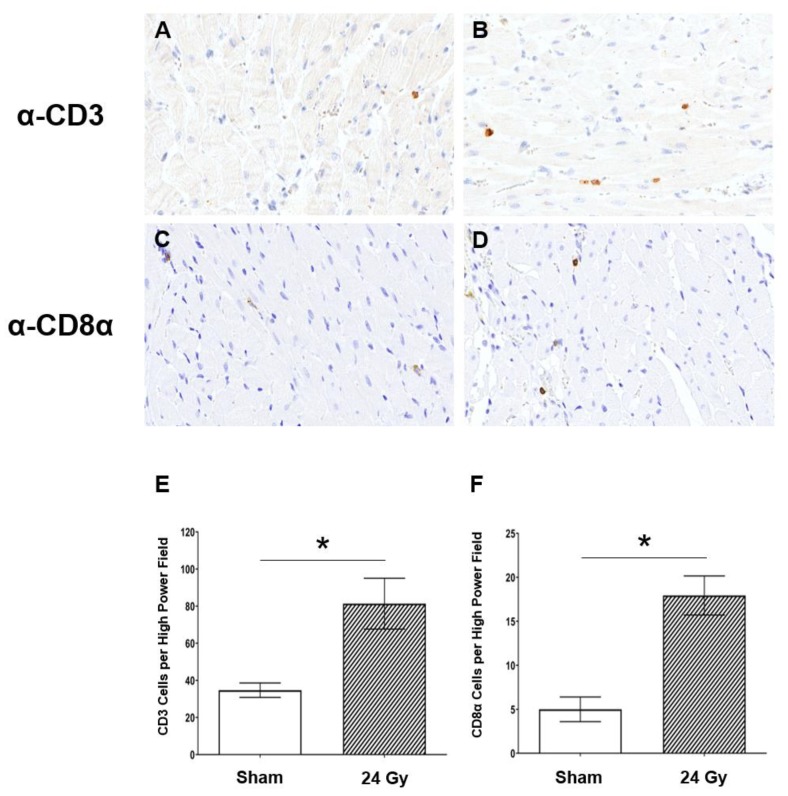
The left ventricles in SS rats have increased T-cell infiltration after localized cardiac radiation compared to sham-treated rats. Adult SS WT female rats were administered either 24 Gy of localized cardiac radiation in one fraction or sham radiation. Representative images of cluster of differentiation 3 (CD3) staining in heart tissue of rats given either (**A**) sham or (**B**) 24 Gy RT. Representative images of CD8α staining in heart tissue of rats given either (**C**) sham or (**D**) 24 Gy RT. T-cell population staining was quantified as the average number of cells per high power field for CD3 (**E**) and CD8α (**F**) stained cell populations (values are means ± standard error of the mean (SEM); *n* = 3–5/group, * *p* < 0.01). All images are at 40× magnification.

Given the increased levels of IL-2 and IL-13 after cardiac RT in female SS WT rats, we next examined whether T cells infiltrated cardiac tissue after RT in SS WT rats. Immunohistochemistry (IHC) was performed on left-ventricular heart tissue of female SS rats administered either sham or 24 Gy × 1 localized cardiac RT and harvested at 10 weeks post-RT. Representative images of CD3 T-cell staining are shown for rats receiving sham treatment (Figure 2A) or 24 Gy cardiac RT (Figure 2B). IHC of a CD3^+^ T-cell subpopulation, CD8α, was also performed, with representative images shown of either sham (Figure 2C) or 24 Gy (Figure 2D). Quantified CD3^+^ and CD8α^+^ cells are presented in Figure 2E,F, respectively. These results demonstrate a statistically significant increase in CD3^+^ cell infiltration, as well as CD8α^+^ cell infiltration, after heart irradiation when compared to sham-treated hearts (*p* < 0.01) at a time when they could contribute to injury in response to cardiac RT. This led us to study the functional role of T cells in promoting radiation-induced cardiotoxicity using a genetic model of immunodeficiency, i.e., the IL2RG^−/−^ SS rat which lacks functional T and B cells and exhibits decreased NK activity [32]. 

### 2.3. The Severity of Pericardial and Pleural Effusions Is Similar between Immune-Competent WT and IL2RG^−/−^ SS Rats

**Figure 3:**
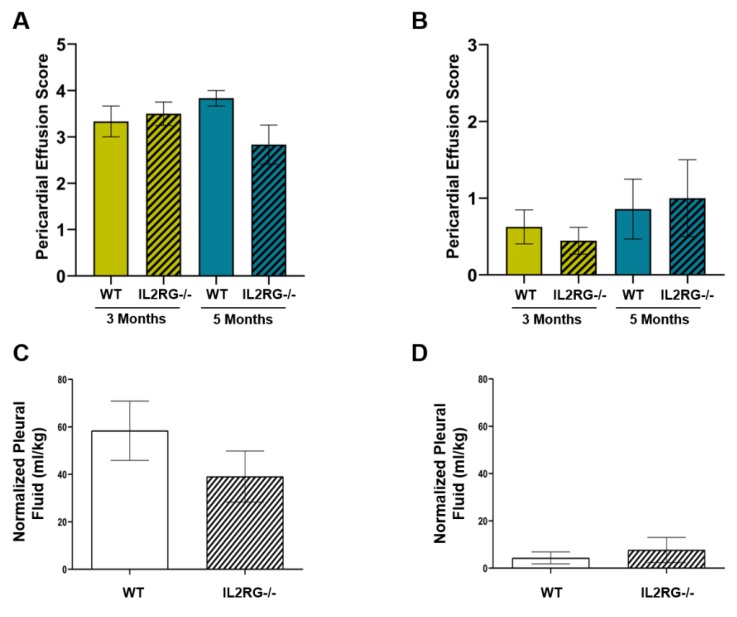
SS interleukin-2 receptor gamma chain knockout (IL2RG^−/−^) rats exhibit no significant differences in pleural or pericardial effusions after 24 Gy localized heart RT treatment, compared with the SS WT immune-competent rats. Adult female rats were treated with 24 Gy of image-guided cardiac radiation therapy (RT). Pericardial effusions were given a score of 0–4 during echocardiogram at three months and five months post-RT for (**A**) females and (**B**) males, with the group average scores displayed. Pleural effusions were collected and quantified at harvest, and normalized to body weight with the (**C**) females having larger amounts of effusion compared to (**D**) males, but no statistical difference was seen between the SS IL2RG^−/−^ and immune-competent SS WT rats. No control rats had pericardial or pleural effusions (not shown). Values are means ± SEM; *n* = 9–15 rats/group. No *p*-values comparing the WT versus IL2RG^−/−^ rats were <0.05.

We previously reported that female SS WT rats receiving 24 Gy × 1 of localized cardiac RT experience pericardial and pleural effusions at three and five months post-RT [26]. Here, we also treated male SS WT rats and SS IL2RG^−/−^ male and female rats with 24 Gy of localized cardiac RT and assessed pericardial and pleural effusion severity and quantity. Some of the SS rats died before the three- and five-month timepoints due to heart failure: two SS WT females before three months, three additional WT females before five months, two SS IL2RG^−/−^ females before five months, and two WT SS males before five months. No male SS IL2RG^−/−^ rats died before the final endpoints. In all surviving rats, pericardial effusion scores were assigned by a sonographer blinded to study groups on a scale of zero (none) to four (large) with the scores defined previously and adapted from clinical recommendations [26,41]. Average effusion scores were graphed for female (Figure 3A) and male (Figure 3B) rats. No statistical differences were observed at either three or five months between WT and IL2RG^−/−^ rats in either sex. Pleural effusions were collected and quantified at the five-month post-RT harvest for female (Figure 3C) and male (Figure 3D) rats. No differences in pleural effusion quantities were observed in IL2RG^−/−^ compared to WT SS rats treated with cardiac RT. Overall, the female rats had higher pericardial effusion scores and quantified pleural effusions compared to male rats, regardless of immune status. These findings demonstrate that pericardial and pleural effusions that accumulate after RT are not T-cell-, B-cell-, or NK-cell-driven, suggesting other mechanisms are responsible for the inflammation and/or heart failure that induce pathological levels of pericardial and pleural effusions [42,43]. Pericardial effusions, where fluid produced by the epicardium accumulates in the pericardial sac, can be markers of pericardial disease and can be caused by inflammation [44,45]. In addition, pleural effusions can be a general indication of heart failure, and they can be also caused by events including radiation pleuritis and/or lymphatic obstruction from mediastinal fibrosis [43,44]. Because effusions are still present in the immunocompromised rats, the potential inflammation causing pericardial effusions is not caused by mature adaptive immune cells including B, T, and NK cells.

### 2.4. Cardiac Hypertrophy Does Not Occur in Female SS IL2RG^−/−^ Rats, Unlike Female SS WT Rats

**Figure 4:**
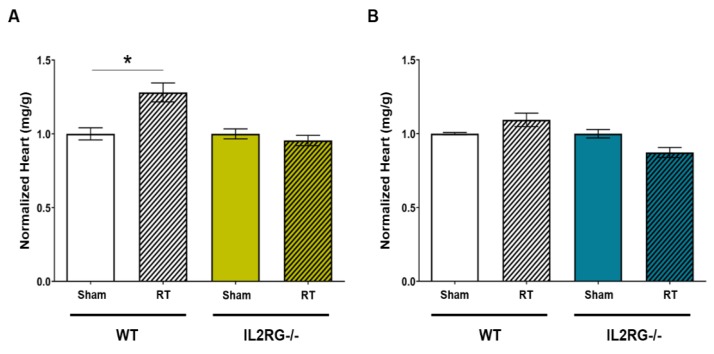
Immune-competent SS WT female rats exhibit cardiac hypertrophy after cardiac radiation, but no hypertrophy occurs in female SS IL2RG^−/−^ rats or in male WT or IL2RG^−/−^ rats. Heart-to-body-weight ratios were measured at harvest in adult SS WT immune-competent and SS IL2RG^−/−^ (**A**) female and (**B**) male rats that were given either sham or 24 Gy RT. At five months, hypertrophy was evident in the female immune-competent SS WT rats that received RT versus sham treatment. At five months, cardiac hypertrophy did not occur in the SS IL2RG^−/−^ female rats. Male rats did not display hypertrophy after 24 Gy (**B**). Values were normalized to the sham-treated animal group average and are the means ± SEM. *n* = 8–15 rats/radiation group and 4–6 rats/control group; * *p* < 0.05.

The WT SS female rats exhibit evidence of left-sided heart failure and cardiac hypertrophy, as measured by normalized heart to body weight ratios, by five months after 24 Gy × 1 localized cardiac RT, as previously reported [26]. Cardiac hypertrophy in SS WT male rats after cardiac RT was not previously reported. We examined whether the SS WT male rats or SS IL2RG^−/−^ male and SS IL2RG^−/−^ female rats exhibited cardiac hypertrophy five months post-24 Gy versus sham RT. The results from the SS IL2RG^−/−^ female rats were compared to previous results from SS WT female rats (Figure 4A) [26]. These results demonstrate that, unlike the SS WT female rats that exhibit cardiac hypertrophy after RT, the SS IL2RG^−/−^ female rats do not exhibit hypertrophy at five months post-RT. The SS WT male rats do not experience statistically significant cardiac hypertrophy six months after RT, nor do the SS IL2RG^−/−^ male rats (Figure 4B). These results demonstrate that, in female SS rats, mature adaptive immune cells are required for the development of cardiac hypertrophy after 24 Gy of localized cardiac RT.

### 2.5. Decreased Heart Function Is Observed via Echocardiogram in SS IL2RG^−/−^ Male and Female Rats at Three Months Post-RT Compared to Immune-Competent SS Rats

**Figure 5:**
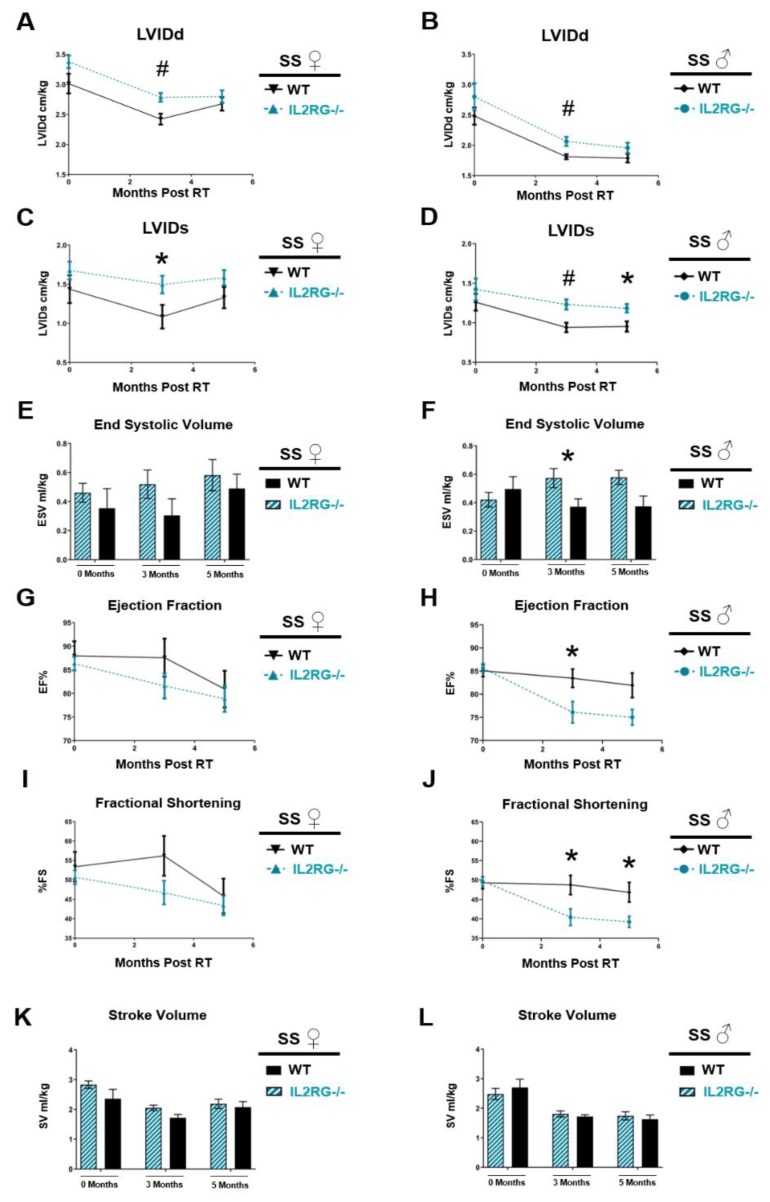
Immune-compromised SS IL2RG^−/−^ male and female rats receiving localized high-dose cardiac RT have echocardiogram parameters revealing worse heart function compared to SS WT rats. SS WT and IL2RG^−/−^ rats were given 24 Gy × 1 localized cardiac RT and echocardiograms were performed, which revealed that the left-ventricular internal diameter at end-diastole (LVIDd) (**A**: female; **B**: male) and at end-systole (LVIDs) (**C**: female; **D**: male) were significantly increased at three months post-RT in SS IL2RG^−/−^ versus SS WT rats, indicating worse function. In male rats, the significantly elevated LVIDs persisted at five months. End-systolic volume (ESV) was not significantly different in female rats, (**E**) but ESV was significantly elevated at three months post-RT in male IL2RG^−/−^ versus WT rats (**F**), which can be associated with worse cardiac function. In females, poorer ejection fraction (EF) (**G**) and fractional shortening (FS) (**I**) values were seen in the IL2RG^−/−^ versus WT rats post-RT (not statistically significant). In males, EF (**H**) and FS (**J**) were significantly decreased in IL2RG^−/−^ rats at three months post-RT, and FS remained decreased at five months, indicating poorer function. Stroke volume (SV) was not significantly different in (**K**) female or (**L**) male rats at any time point. Values are means ± SEM; *n* = 9–15 rats/group, * *p* < 0.05, # *p* < 0.01.

The SS WT male and female rats were previously reported to manifest echocardiograph changes consistent with the development of left-sided heart failure at 3–6 months post-RT [26]. To compare the echocardiograph changes in rats lacking functional T cells, the IL2RG^−/−^ female and male rats were treated with 24 Gy × 1 or sham as described above, and echocardiograms were performed. Multiple echocardiograph parameters indicated that the SS IL2RG^−/−^ female and male rats had poorer function compared to the SS WT immune-competent rats [26] at three months (females and males) and five months (males) post-RT (Figure 5, Tables S1 and S2). Left-ventricular internal diameter at end-diastole (LVIDd, Figure 5A,B) and at end-systole (LVIDs, Figure 5C,D) were significantly increased in SS IL2RG^−/−^ male and female rats at three months post-RT compared to WT SS rats, indicating poorer heart function, with LVIDs remaining significantly higher in the IL2RG^−/−^ male rats at five months post-RT. End-systolic volume (ESV) had a trend toward higher values, which was not statistically significant, in the SS IL2RG^−/−^ compared to SS WT female rats (Figure 5E). However, ESV was significantly elevated in the SS IL2RG^−/−^ compared to the SS WT male rats at three months post-RT (Figure 5F), also indicating poorer cardiac function in the SS IL2RG^−/−^ male rats compared to male SS WT rats.

Ejection fraction (EF, Figure 5G,H, females and males, respectively) and fractional shortening (FS, Figure 5I,J, females and males, respectively) were significantly decreased in the male SS IL2RG^−/−^ compared to SS WT rats. Specifically, EF values were significantly decreased in the SS IL2RG^−/−^ versus SS WT male rats three months post-RT, and EF remained decreased at five months, but this difference was not statistically significant (Figure 5H). FS percentages were significantly decreased in the SS IL2RG^−/−^ versus SS WT male rats at both three and five months post-RT. Taken together, the decreases in EF and FS in the IL2RG^−/−^ male rats indicate poorer cardiac function (Figure 5J). EF and FS values in female SS IL2RG^−/−^ rats tended to be lower than in SS WT female rats, but these differences were not statistically significant (Figure 5G,I). Lastly, stroke volume (SV), the amount of blood pumped out of the left ventricle in each contraction, was not significantly different between SS WT and IL2RG^−/−^ rats (Figure 5K female, Figure 5L male). These results reveal that, as assessed by echocardiogram, cardiac injury in response to a large, localized, single-dose fraction of RT can actually be made worse by the loss of adaptive immune cells when compared to immune-competent rats.

**Figure 6:**
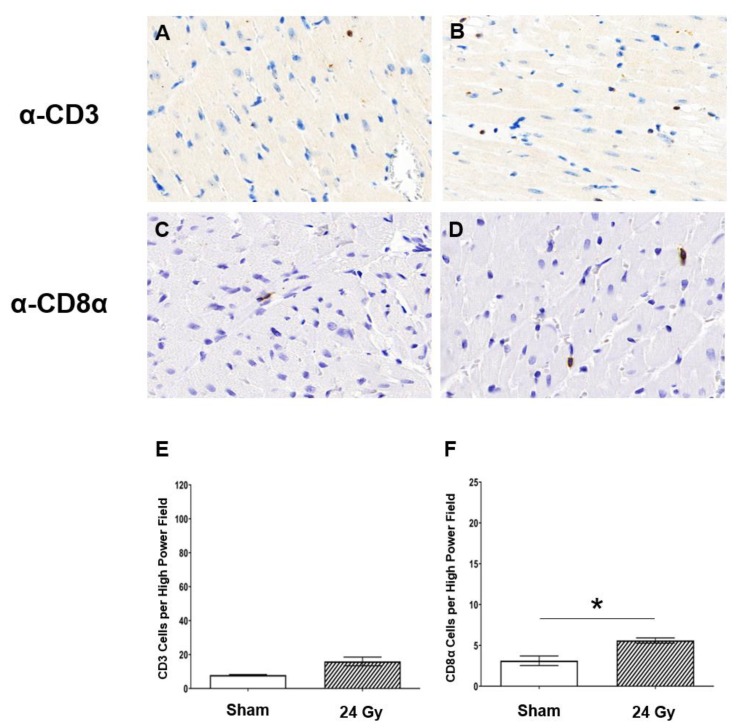
Irradiated SS WT male rat hearts display trends of increased T-cell infiltration compared to sham-treated rats, but have less T-cell infiltration than SS WT female rats. Adult SS WT male rats were administered either 24 Gy of localized cardiac radiation in one fraction or sham radiation. Representative images of CD3 staining in heart tissue of rats given either (**A**) sham or (**B**) 24 Gy RT. Representative images of CD8α staining in heart tissue of rats given either sham (**C**) or 24 Gy RT (**D**). T-cell population staining was quantified as the average number of cells per high-power field for CD3 (**E**, *p* = 0.057 for sham versus 24 Gy) and CD8α (**F**) stained cell populations (values are means ± SEM; *n* = 3–5/group, * *p* < 0.01). Values are means ± SEM; *n* = 3–5 rats/group, * *p* < 0.05. All images are at 40× magnification.

The male versus female rats display some differences in the way that the SS IL2RG^−/−^ versus WT rats respond to high-dose, single-fraction RT to the heart (Figure 3 and Figure 5). Thus, we examined the infiltration of CD3-positive and CD8α-positive cells into the heart at 10 weeks after RT or sham treatment in male SS WT rats (Figure 6), using similar methods to those used in female SS WT rats in Figure 2. This revealed a trend toward an increase in CD3-positive cells in the heart after RT (Figure 6A,B,E), and a small but significant increase in CD8α-positive cells after RT (Figure 6C,D,F). However, comparing the numbers of CD3-positive or CD8α-positive cells after RT in males (Figure 6) versus females (Figure 2; graphs have the same scales) revealed a marked increase in the number of CD3-positive cells in female versus male SS WT rat hearts with and without RT treatment (sham-treated 34.70 ± 3.91 female versus 7.92 ± 0.36 male, *p* < 0.01; 24 Gy RT 81.33 ± 13.68 female versus 15.97 ± 2.56 male, *p* < 0.001, Figure 2E versus Figure 6E). In addition, CD8α-positive cells were markedly increased after 24 Gy RT in female versus male rat hearts (17.94 ± 2.22 female versus 5.60 ± 0.32 male, *p* < 0.001, Figure 2F versus Figure 6F). The increase in T-cell populations in female compared to male rat hearts may in part contribute to sex differences observed in the severity of developing RIHD. 

**Figure 7:**
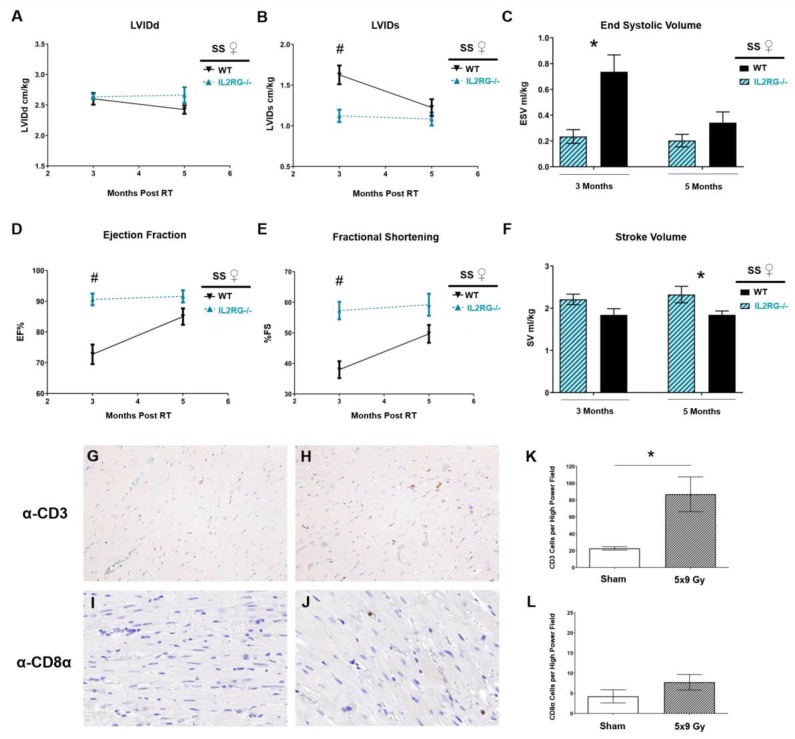
SS IL2RG^−/−^ immune-compromised rats versus SS WT immune-competent rats receiving fractionated 9 Gy × 5 cardiac radiation have slightly less cardiac damage measured via echocardiogram. Female SS IL2RG^−/−^ and SS WT rats received 9 Gy × 5 localized cardiac radiation, and echocardiograms were performed at three and five months post-RT revealing that (**A**) left-ventricular internal diameter at end-diastole (LVIDd) was not significantly different between the groups at three or five months after 9 Gy × 5. (**B**) LVID at end-systole (LVIDs) and (**C**) end-systolic volume (ESV) were significantly lower in the SS IL2RG^−/−^ rats versus SS WT rats at three months, which can be associated with improved cardiac function. The (**D**) ejection fraction (EF) and (**E**) fractional shortening (FS) were increased in SS IL2RG^−/−^ versus SS WT rats, indicating improved function. However, these changes did not persist at five months post-RT. (F) Stroke volume (SV) was significantly elevated at five months post-RT in the IL2RG^−/−^ rats compared to WT, also indicating improved cardiac function. Values are means ± SEM; *n* = 11/WT and *n* = 6/IL2RG^−/−^; * *p* < 0.05, # *p* < 0.01. Representative images of CD3 staining in heart tissue of rats given either (**G**) sham or (**H**) 5 × 9 Gy RT. Representative images of CD8α staining in heart tissue of rats given either sham (**I**) or 5 × 9 Gy RT (**J**). Cardiac T-cell infiltration was quantified as the average number of cells per high-power field for CD3-positive (**K**) and CD8α-positive (**L**, *p* = 0.29 for comparison of sham versus 9 Gy × 5) cell populations (values are means ± SEM, *n* = 3–6/group, * *p* < 0.05). All images are at 40× magnification.

We subsequently expanded our study to a more clinically relevant fractionated RT dose, using an image-guided localized cardiac RT regimen of 9 Gy × 5 daily fractions [26,46,47,48]. This regimen was previously shown to cause RIHD in the SS WT rats [26]. We treated SS IL2RG^−/−^ female rats with 9 Gy × 5, using identical beam arrangements and weighting as in the 24 Gy × 1 treatment [26]. Echocardiograms were performed at three and five months post-RT, and values from SS WT and SS IL2RG^−/−^ rats were compared (Figure 7). Interestingly, treatment of SS IL2RG^−/−^ rats with 9 Gy × 5 caused less cardiac damage than in the SS WT female rats, as measured by statistically significantly decreased LVIDs (Figure 7B) and ESV (Figure 7C), and increased EF (Figure 7D) and FS (Figure 7E) at three months post-RT in the SS IL2RG^−/−^ rats. While these parameters did not remain statistically different at five months post RT, SV was statistically increased in the SS IL2RG^−/−^ rats at five months post-RT (Figure 7F), which is also an indicator of improved cardiac function in the SS IL2RG^−/−^ versus SS WT rats. These changes are distinct from the worsening of cardiac function seen in the SS IL2RG^−/−^ versus SS WT rats treated with 24 Gy × 1 (Figure 5). T-cell infiltration of CD3-positive and CD8α-positive cells into the heart at 10 weeks after 9 Gy × 5 fractions of RT or sham treatment revealed increased CD3-positive staining after 9 Gy × 5 RT versus sham (*p* < 0.05, Figure 7G,H,K). However, there was no significant difference in CD8α-positive stained cells after 5 × 9 Gy RT compared to sham treatment (*p* = 0.29, Figure 7I,J,L). These results demonstrate that both 24 Gy × 1 and 9 Gy × 5 cardiac RT regimens cause CD3-positive T-cell recruitment to the damaged heart, while CD8α-positive T cells were only increased with the 24 Gy RT treatment (Figure 2E,F versus Figure 7K,L). These results highlight the complex role that acquired immune cells can play in radiation-induced cardiac injury depending on treatment characteristics.

### 2.6. Decreased Heart Function Observed in only IL2RG^−/−^ Male SS.BN3 Rats Compared to Immune-Competent SS.BN3 Rats after High-Dose Single-Fraction Cardiac RT

**Figure 8:**
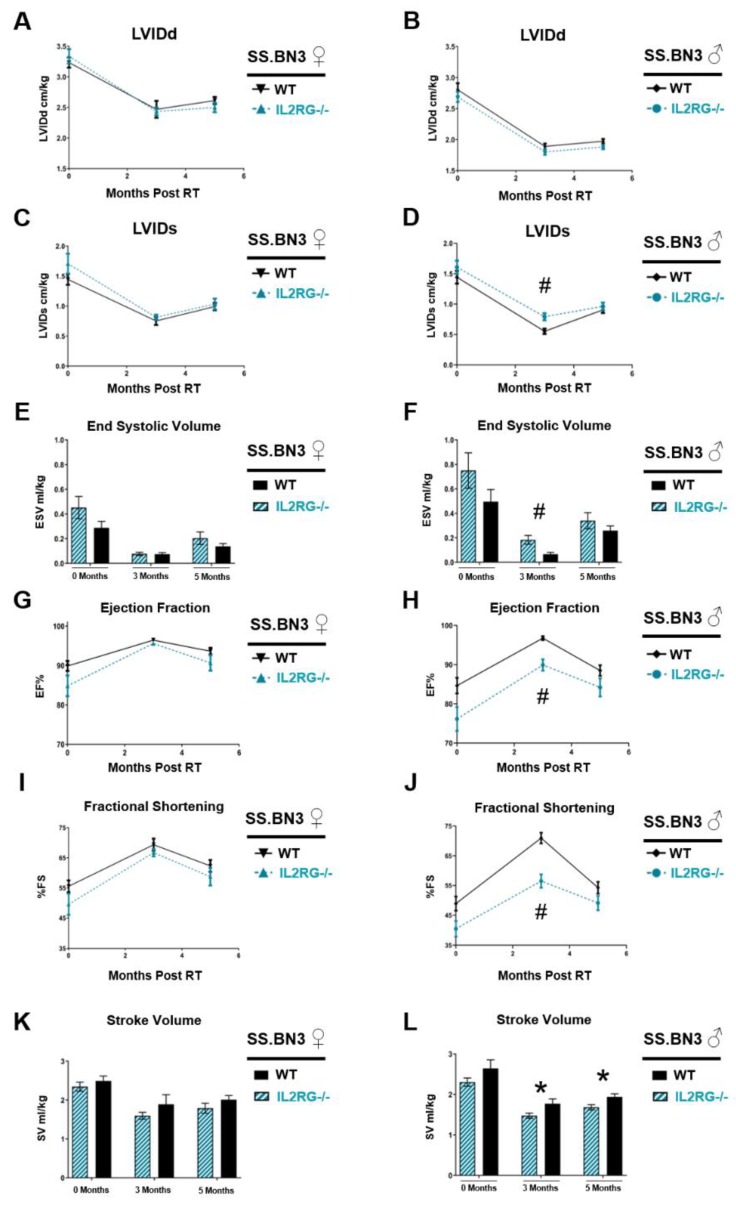
The consomic SS.BN3 rats exhibit some differences in cardiac function from the SS rat strain when comparing immune-compromised (IL2RG^−/−^) versus WT rats after single-dose cardiac RT. After receiving 24 Gy of localized cardiac RT, only the male SS.BN3 IL2RG^−/−^ rats had echocardiogram parameters indicating poorer heart function, while both male and female SS IL2RG^−/−^ rats had parameters indicating poorer cardiac function compared to WT SS rats (Figure 5). The left-ventricular internal diameter at end-diastole (LVIDd) (**A**: female; **B**: male) was not significantly different in SS.BN3 IL2RG^−/−^ versus SS.BN3 WT rats, and LVIDs (**C**: female; **D**: male), had significantly decreased values in IL2RG^−/−^ rats only in males at three months post-RT (**D**). End-systolic volume (ESV) was not significantly different in female (**E**) but was increased at three months post-RT in male (**F**) SS.BN3 IL2RG^−/−^ versus SS.BN3 WT rats. In females, ejection fraction (EF) (**G**) and fractional shortening (FS) (**I**) were not significantly different, whereas, in males, EF (**H**) and FS (**J**) were significantly decreased at three months post-RT in the SS.BN3 IL2RG^−/−^ rats, indicating poorer cardiac function. Stroke volume (SV) was not significantly different in (**K**) female but was in (**L**) male rats at three and five months post-RT, with statistical decreases in the SS.BN3 IL2RG^−/−^ versus SS.BN3 WT rats, also suggesting worse cardiac function. Values are means ± SEM; *n* = 7–13 rats/group, * *p* < 0.05, # *p* < 0.01.

We previously demonstrated the importance of genetic background for cardiac injury in this model using a consomic chromosome substitution approach, where inbred SS rats have chromosome 3 inherited from the BN strain, with the remainder of the chromosomes from the SS strain (SS.BN3). This SS.BN3 rat strain is more resistant to localized cardiac RT than the parental SS strain [26]. Therefore, genetic variants on rat chromosome 3 inherited from the BN strain provide protection against cardiac RT compared to the SS WT rats. To examine whether genetic background alters the effects of adaptive immune cells on RIHD, male and female SS.BN3 IL2RG^−/−^ rats were administered localized 24 Gy cardiac RT, as described above, and echocardiograms were performed at baseline, as well as at three and five months, to monitor heart function over time. The echocardiogram parameters in male and female SS.BN3 IL2RG^−/−^ rats and male and female SS.BN3 WT rats [26] are shown in Figure 8. In females, LVIDd (Figure 8A), LVIDd (Figure 8C), ESV (Figure 8E), EF, and FS (Figure 8G,I), as well as SV (Figure 8K), were not significantly different at zero, three, or five months post-24 Gy between the SS.BN3 IL2RG^−/−^ and SS.BN3 WT rats. However, multiple echocardiograph parameters indicated that the SS.BN3 IL2RG^−/−^ male rats had poorer function compared to the SS.BN3 WT immune-competent rats, similar to results seen in the SS rat strain **(**Figure 5). LVIDd was not significantly different in SS.BN3 IL2RG^−/−^ versus SS.BN3 WT male rats (Figure 8B), but LVIDs was statistically increased at three months post-RT in IL2RG^−/−^ males (Figure 8D). ESV was also significantly elevated at three months post-RT in IL2RG^−/−^ versus WT SS.BN3 male rats (Figure 8F), which coincided with decreased EF (Figure 8H) and FS (Figure 8J) in IL2RG^−/−^ rats compared to SS.BN3 WT immune-competent rats. Lastly, SV was significantly elevated at three and five months post-RT in the IL2RG^−/−^ versus WT SS.BN3 male rats. Cardiac injury varied between rat strains SS and SS.BN3 in a manner that depended on sex. Male SS.BN3 results were similar to those seen in male SS rats, whereas the IL2RG^−/−^ group exhibited poorer heart function after RT. However, while female SS rats deficient in IL2RG had statistically increased LVIDd and LVIDs at three months post-RT compared to SS WT rats (Figure 5), the SS.BN3 female rats displayed no differences with immunodeficiency (Figure 8). These data highlight the complicated role of adaptive immunity, sex, and genetic backgrounds in RIHD.

## 3. Discussion

In the current study, we demonstrate altered development of RIHD after localized radiation exposure to the heart in immune-competent SS WT rats compared to immunodeficient SS IL2RG^−/−^ rats. We analyzed the role of acquired immune cells, accounting for clinically relevant parameters including RT dose and fractionation schemes, sex, and genetic background using closely related consomic strains with previously identified changes in susceptibility to injury. Our results reveal a complex role for adaptive immune cells in the development and severity of RIHD that is highly dependent on these important contexts. We show that mature T cells are not universally required for the development of RIHD, and that the loss of adaptive immune cells can even worsen important parameters of cardiac function as assessed by echocardiogram in SS IL2RG^−/−^ versus SS WT rats receiving a high single dose of cardiac RT (Figure 5). In contrast, immunodeficiency led to improvement in some cardiac parameters in rats receiving fractionated 9 Gy × 5 localized cardiac RT (Figure 7). In general, changes between the SS IL2RG^−/−^ and SS WT rats were more pronounced in male versus female rats. In addition, the differences were dependent upon the genetic background of the treated rats (Figure 8).

Adaptive immune cells are known to alter cardiac function. After insults such as myocardial infarction or infection, the immune system facilitates removal of dead tissue [20], but immune responses can also cause adverse tissue remodeling leading to irreversible damage [20,21]. In particular, effector mechanisms (e.g., perforin/granzyme and Fas/FasL interactions) can be triggered from cytotoxic T cells interacting with a target cells, which can alter cardiomyocyte function and lead to perturbation of the cardiac remodeling process [49,50]. Lymphocytes are a common component of the leukocyte infiltration that occurs in tissues after irradiation [49,50,51], and T cells can both promote and protect against adverse outcomes in tissue depending on a wide range of factors [52]. In our model of localized cardiac RT, there were elevated levels of IL-2 and IL-13 in SS WT rat plasma after localized high-dose cardiac RT (Figure 1). IL-2 binds the IL-2 receptor, which is important for the maintenance of several lymphoid populations, including activated effector T cells, regulatory T cells (Tregs), memory T cells, and natural killer (NK) cells [51]. IL-13 is generally thought of as a T helper type 2 (Th2) cytokine involved in a variety of disorders, including cancer and inflammatory disease. Some studies indicated that IL-13 may be produced by additional T-cell subsets; however, once produced, it binds the IL-4/IL-13 receptor complex, where it exerts important effects on inflammation, tissue remodeling, and fibrosis [27,38,39]. We, therefore, determined whether T cells were recruited into the irradiated cardiac tissue at a time (10 weeks) that could be significant for the injury we observe post-RT. We detected increased CD3^+^ (all T cells) and CD8α^+^ T-cell infiltrates in the left-ventricular tissue of SS immune-competent WT rats receiving high-dose cardiac RT (Figure 2), which led us to further examine the role of these important immune cells in RIHD.

Here, we utilized many parameters to assess the severity of RIHD in our rat model, and many of the changes seen in the rats after cardiac RT are relevant to the post-RT cardiac dysfunction that can be seen in humans. This study utilized high doses of RT to the heart. While these exact doses and fractionation schemes are not received clinically, high doses of fractionated radiation to portions of the heart are received by some patients as part of their cancer treatments. For example, in one study of 190 non-small-cell lung cancer patients receiving three-dimensional (3D) conformal RT, the volume of the heart receiving ≥50 Gy (V50) was a median of 25%, with the 75th percentile for the heart V50 of 39%. The heart V60 for these patients was 17% (75th percentile for the heart V60 was 28%) [53]. Other studies of stereotactic body radiation therapy (SBRT) for lung cancers, using a linear quadratic model to approximate equivalent doses, with an estimated α/β ratio of 2 for the heart, demonstrated that the maximum dose to the heart was the equivalent dose at 2 Gy (EQD2) of >200 Gy for some patients [54,55]. For our rat models of RIHD, the single- and multi-fraction regimens previously showed similar cardiovascular dysfunction, which also recapitulates the dysfunction that can be seen in patients. In our rat model, phenotypes indicative of RIHD included hypertrophy (Figure 3), pericardial and pleural effusions (Figure 4), and mortality (data not shown) [26]. Additionally, echocardiogram parameters indicative of heart dysfunction were elevated LVIDd, LVIDs, ESV, and EDV, and decreased EF and FS (Figure 5). The most common types of cardiac dysfunction seen in patients after cardiac RT exposure include pericarditis, pericardial and myocardial fibrosis, coronary artery disease, and/or conduction and valvular abnormalities [1,56]. Wang et al. assessed the types of cardiac events in patients treated on dose-escalation trials for stage III non-small-cell lung cancer. Pericardial (pericardial effusions or pericarditis), ischemic (myocardial infarction or unstable angina), and arrhythmic (significant arrhythmia) events were correlated with whole-heart doses and other parameters [57]. Ventricular dysfunction from cardiac RT can also lead to systolic or diastolic function. The effusions, cardiac hypertrophy, and left-ventricular echocardiogram changes observed in our rat model are relevant to human RIHD, particularly the pericardial events and ventricular dysfunction. Thus, our studies using the IL2RG^−/−^ rat model to examine the contribution of T, B, and/or NK cells in RIHD have relevance to many of the manifestations of RIHD observed in patients after RT exposure.

Mice with *IL2rg* gene disruption have x-linked severe combined immunodeficiency (X-SCID), with the immunodeficiency characterized by a profound loss of T cells, B cells, and NK cells [29,30,31], and IL2RG^−/−^ rats display similar immune cell deficiencies [32]. Here, we utilized SS IL2RG^−/−^ rats [33,34] as a rat model lacking mature B and T cells. Using the SS IL2RG^−/−^ SS rats, we observed an enhanced sensitivity to 24 Gy localized cardiac RT when compared to SS WT rats, resulting in increased cardiac toxicity (Figure 5). Sex differences were also observed in the IL2RG^−/−^ versus WT SS rats. Female rats receiving RT, regardless of immune status, had higher mortality rates than male rats [26] and increased severity of pericardial and pleural effusions when compared to male rats receiving RT (Figure 3). Cardiac hypertrophy was observed in female immune-competent rats only, compared to male competent, or male or female IL2RG^−/−^ rats (Figure 4). The differences in the severity of RIHD may be partly explained by body size and the percentage of total lung tissue exposed to high doses of RT. All rats were aged 10–12 weeks at the time of RT, but the male rats were larger than females with increased body weight (295.1 ± 11.6 g versus 194.0 ± 3.7 g, *p* < 0.001). During computed tomography (CT) scans for RT planning, we observed that the female rats had smaller total lung volumes compared to male rats (not shown); thus, the females rats have a higher percentage of lung receiving high-dose RT than age-matched males. Other than—or in addition to—these differences, there may also be differences in the response to cardiac RT exposure between males and females. Bates et al. analyzed the risk for late-onset cardiac diseases in survivors of childhood cancer given RT and/or anthracyclines and reported that female survivors were at greater risk of heart failure, most likely due to anthracycline-related heart failure, and male survivors had a slightly increased risk of coronary artery disease, with an unclear explanation for these observed sex differences [58]. Mulrooney et al. found that, in childhood cancer survivors, on multivariate analysis adjusting for cardiac radiation doses, anthracycline doses, and other factors, females were more likely to experience heart failure after cancer treatment [59].

In this study, we utilized a single fraction of high-dose RT, in addition to a more clinically relevant fractionated radiation regimen of 9 Gy × 5 fractions. Using the 9 Gy × 5 regimen, the IL2RG^−/−^ rats had slightly decreased sensitivity to RT, resulting in improved cardiac parameters such as EF and FS at three months post-RT, when compared to the immune-competent SS WT rats (Figure 7). Using a linear quadratic model to approximate equivalent doses, with an estimated α/β ratio of 3 for the heart (although it is difficult to model cardiac equivalent doses using this model due to the complexity of the structure of the heart) [60], the 9 Gy × 5 regimen yields an equivalent dose at 2 Gy fractions of approximately 108 Gy, while the 24 Gy × 1 regimen yields an equivalent dose at 2 Gy fractions of approximately 130 Gy. While it is also difficult to model equivalent doses for large fraction sizes and with single-fraction regimens, our previous results indicated that the 24 Gy × 1 regimen yields cardiotoxicity that is either equivalent to or worse than that sustained in rats receiving 9 Gy × 5 [26]. Comparing immune-competent SS female rats that received 24 Gy × 1 to those receiving 9 Gy × 5 fractions, we did not see differences in mortality [26], the presence of moderate to large pericardial effusions, or the increase in CD3-positive cells infiltrating the heart after RT (81.33 ± 13.68 with 24 Gy, Figure 2E and 86.88 ± 20.73 with 9 Gy × 5, Figure 7K). In addition, both radiation regimens led to cardiac hypertrophy compared to their respective sham-treated rats [26]. Pleural effusions in SS rats receiving 24 Gy had significantly higher volumes compared to effusions in rats receiving 9 Gy × 5 fractions (12.9 ± 2.5 mL versus 5.4 ± 1.9 mL, *p* < 0.05 [26]). Echocardiogram parameters between the SS female rats receiving 24 Gy × 1 and 9 Gy × 5 fractions both demonstrated evidence of ventricular dysfunction compared to sham-treated controls [26]. It is not clear why the SS IL2RG^−/−^ rats treated with 9 Gy × 5, when compared to the SS WT rats receiving 9 Gy × 5, did not show similarly decreased cardiac function changes to the results seen in SS IL2RG^−/−^ versus SS WT rats treated with 24 Gy × 1 (Figure 5 versus Figure 7). This could be due to differences in adaptive immune system involvement and regulation that are dependent upon the total dose of radiation received, or due to the radiation fraction sizes and the number of fractions of radiation received. Very few studies examined the signaling and molecular changes that occur after single versus fractionated cardiac RT. In tumor stroma studies, tumor cell apoptosis can be associated with low doses of RT, where necrotic cell death is associated with higher doses [61]. While the apoptotic cell death is viewed as both tolerogenic and immunogenic, necrotic death can also be considered immunogenic if the process is accompanied with the release of stress signals [61]. However, it is unclear how findings from tumor studies relate to normal tissue toxicity in the heart. With fractionated dose regimens, recruited leukocytes would also be subject to radiation damage, and they serve as a potential source of pro-inflammatory “danger signals” due to cell death. Lymphocytes are a common component of leukocytic infiltrates after irradiation [52,62,63], and T-cell responses can either drive or protect against normal tissue side effects [64]. Klug et al. reported that low-dose RT redirects macrophage differentiation to an M1 subtype that enables the recruitment of cytotoxic T cells compared to the immune-suppressive M2 subtype associated with high-dose RT [65]. The worse effects observed in the WT versus IL2RG−^/−^ rats with 5 × 9 Gy RT may be due to the ability of WT rats to recruit T cells to potentially exacerbate damage, where this response is not possible in IL2RG^−/−^ rats (Figure 7). These changes could have important consequences for the overall inflammatory response, indicating the need for further studies to determine the most advantageous dosing regimens for treatments directed at both the tumor and the patient’s immune response.

We previously demonstrated that genetic variants can greatly alter the sensitivity of rats to cardiac radiation exposure [26]. These studies demonstrated that the inbred SS rat strain is very sensitive to localized cardiac radiation exposure when compared to the Brown Norway (BN) strain or the SS rats with chromosome 3 substituted from the BN strain (consomic SS.BN3 rats) [26]. Our results indicate that the lack of adaptive immune cells caused by IL2RG deficiency leads to worsening of some cardiac parameters in rats treated with 24 Gy × 1 in a manner that is dependent on the sex and genetic background of the host. To examine whether genetic changes can alter RIHD in IL2RG^−/−^ rats, we utilized the consomic SS.BN3 IL2RG^−/−^ rats, comparing the cardiac function to SS.BN3 WT rats after cardiac RT. While the male SS.BN3 IL2RG^−/−^ rats demonstrated worse cardiac function when compared to immune-competent SS.BN3 rats, the female IL2RG^−/−^ SS.BN3 rats did not (Figure 8). In SS rats, some cardiac parameters indicating worse dysfunction were also seen in the SS.BN3 IL2RG^−/−^ rats (Figure 5). Interestingly, these results also indicate that adaptive immune cells cannot account for our previously reported differences in radiation sensitivity between SS and SS.BN3 rats [26], as echocardiogram data demonstrate that IL2RG-deficient SS.BN3 rats are still significantly protected against RIHD when compared to the IL2RG-deficient SS rats (compare Figure 5 and Figure 8, direct comparison data not shown). Taken together, these studies highlight the importance of sex and host genetic background in the interpretation of results from normal tissue biology studies.

Recent advances in combinational cancer therapies, where the patient may receive radiation, as well as immunotherapy, hold great promise. In light of this, there is increasing interest in understanding how immunotherapy, including checkpoint inhibitors, could alter the effects of RT on the heart [66,67]. Radiation can alter immune responses, and the effects of radiation can change based on the state of both adaptive and innate immunity. The effects of radiation on tissue or immune cells, directly, could influence immunotherapy, with the potential to exacerbate tissue injury. Furthermore, many cancer patients who receive RT also receive systemic therapies such as chemotherapy. While there were studies to investigate the effects of combining cardiac RT and chemotherapeutic agents such as doxorubicin [68,69,70] and sunitinib [48], the cardiac effects of the combination of radiation and immunotherapies are less studied [66,67]. Because many immunotherapies target the mechanisms that restrain T cells to enhance tumor immunity, such as blockade of cytotoxic T-lymphocyte-associated protein 4 (CTLA-4) with ipilimumab [71] or programmed death protein 1 (PD-1) with pembrolizumab [72], these approaches could adversely impact normal cardiac tissue [66,67,71,72]. Du et al. demonstrated with C57Bl/6 mice that whole-heart RT with PD-1 blockade led to increased mortality and cardiac dysfunction compared to mice that were only given cardiac RT [66]. With respect to the immune cell subtypes present, Myers and Lu saw increased mortality, as well as an increase in number of T cells, in the lungs and heart of C57Bl/6 mice that received thoracic RT and an anti-PD-1 antibody compared to mice given thoracic RT [67]. These findings suggest an enhanced sensitivity to cardiac RT when the immune system, specifically cytotoxic T lymphocytes, is no longer suppressed.

In this study, the well-established *IL2rg* gene disruption model of immune deficiency allowed us to study the contributions of T cells to the development of RIHD. However, there are limitations to this model that should be taken into account when interpreting results. The loss of T cells is not the only difference, since B and NK cell functions are also diminished. These defects could influence normal tissue toxicity to RT [18,73,74]. Nonetheless, these results demonstrate that T cells are not uniformly required for the development of injury in our model. Despite this, immunodeficiency in this model alters the response to cardiac radiation exposure, as demonstrated by changes in cardiac injury that differ based upon clinically relevant parameters such as RT dose and fractionation, the sex of the subject, and genetic background. There are a variety of future studies that will shed additional light on how the immune system components influence sensitivity to cardiac RT, such as utilizing knockout animal models for T-cell subpopulations (e.g., *FOXP3* for regulatory T cells). Additionally, mature B and NK cells are absent in our IL2RG^−/−^ rat model. Antibodies could also be utilized to target other immune cell populations (e.g., rituximab to deplete B cells). Finally, additional studies more clinically relevant cardiac exposures, such as partial heart radiation with smaller fraction sizes, will be important to shed light on the role of adaptive immunity in RIHD patients. These results highlight the need for additional studies to better understand how immune modification due to cancer therapies can alter RIHD development in patients.

## 4. Materials and Methods

### 4.1. Rat Model and Irradiation Procedure

SS and SS.BN3 rats with a disrupted *IL2rg* gene (IL2rg^em2MCWi^, MCW Rat Genome Database), created as described previously [33,34], were utilized for this study. The rat experimental cardiac radiation procedure was reported elsewhere [26]. In brief, male and female IL2RG^−/−^ SS rats and Salt-Sensitive/Brown Norway consomic SS.BN3 rats (Medical College of Wisconsin) aged 10–12 weeks were randomized into different treatment groups [26]. Animals were anesthetized with 3% isoflurane with inhaled room-temperature air and administered localized heart irradiation using a the high-precision image-guided X-RAD SmART irradiator (Precision X-Ray, North Branford, CT, USA). The irradiator output was regularly verified using a calibrated ionization chamber. Either 24 Gy × 1 or 9 Gy × 5 fractions were administered by placing isocenter in the center of the heart, with equally weighted anterior–posterior and two lateral beams using a 1.5-cm collimator (1:1:1, 225 kVp, 13 mA, 0.32 mm Cu, 2.69 Gy/min). The position of the heart was determined by creating computed tomography scans in transverse, sagittal, and frontal views. With each projection, the heart was centered to fit into the collimator. The whole heart was included in the irradiated volume. Radiation doses were calculated using Monte-Carlo-based treatment planning (MAASTRO Radiotherapy Clinic, Netherlands). Age-matched sham-irradiated (0 Gy) animals were included in the study. The immune-competent SS and SS.BN3 rat results were previously reported [26]. Animals were irradiated and housed at the Medical College of Wisconsin with access to a standard diet (0.4% salt) and reverse-osmosis hyperchlorinated (between 2–3 ppm) water, with a 12-h/12-h light/dark cycle and sterile pathogen-free conditions in single ventilated cages with hardwood chip bedding material. Adult male rats were housed with three or fewer animals/cage, while adult female rats were housed with five or fewer animals/cage. Studies were designed in accordance with Animal Research: Reporting of In Vivo Experiments (ARRIVE) guidelines [75]. All procedures for this study were approved by the Institutional Animal Care and Use Committee of the Medical College of Wisconsin and were performed according to the American Guidelines for the Ethical Care of Animals (ethical code: AUA 4200, approved on 5 September 2018).

### 4.2. Rat Cytokine Array

Rat blood was drawn from the right ventricle during harvest at one and 10 weeks post-RT. The needle was coated with ethylenediaminetetraacetic acid (EDTA) to prevent coagulation. Blood samples were immediately subjected to a series of centrifugations to isolate the plasma and stored as aliquots at −80 °C. Plasma aliquots were used to perform a rat cytokine array, with sample conditions blinded (Chemokine Array RD 27; Eve Technologies, Calgary, AB, Canada).

### 4.3. Echocardiography

The rat echocardiogram procedure was reported elsewhere [26]. In brief, cardiac function was assessed using echocardiography with M-mode readings on irradiated and sham-treated rats at baseline, three or five months post-RT. An echocardiograph Vivid 7 (General Electric, Wauwatosa, WI, USA) was used, with an 11-MHz M12L linear-array transducer and EchoPac software (General Electric, Wauwatosa, WI, USA). Imaging was conducted in the short-axis view at the mid-level of the left ventricle, by a sonographer blinded to study treatment groups. Three consecutive heartbeats were measured, and the average was utilized for analyses [76,77], with the imaging and analysis performed as previously described [26]. Echocardiogram parameter values (except for EF and FS, which are a percentage) were normalized to body weight [27]. The amount of effusion was given a score from 0–4, and the average scores of the group were determined. Some of the SS rats died of heart failure before the echocardiogram timepoints due to heart failure: two SS WT females before three months, three additional WT females before five months, two SS IL2RG^−/−^ females before five months, and two WT SS males before five months. No SS.BN3 rats died before the final endpoints. All echocardiograms were performed in the morning hours, with planned animal harvests following echocardiograms. Irradiation, echocardiograms, and collection of heart specimens were performed within the animal facility.

### 4.4. Histological Analysis

Hearts were excised and fixed in zinc formalin at harvest (five months post-RT) or at time of death from those that died from heart failure due to treatment prior to the five-month time point. Tissues were fixed for 48 h and then transferred to 70% ethanol. Tissue processing and immunohistochemical analyses were reported previously [26]. For T-cell staining, anti-CD3 (ab16669, Abcam, Cambridge, UK) and anti-CD8α (ab33786, Abcam, Cambridge, UK) were used to detect immune cell populations on heart tissue at 10–11 weeks post-RT. All images were acquired using a Nikon Eclipse 50i upright microscope equipped with a Nikon Digital sight DS-U3 camera and NIS Elements BR software (Nikon Instruments, Melville, NY, USA). CD3 and CD8α T-cell staining was quantified by an automated count to detect the number of cells per high-power field above the set threshold or counted by counting the number of cells per high-power field on blinded samples as previously reported, with the observer blinded to the experimental conditions [26]. 

### 4.5. Statistical Analysis

Analyses of the echocardiogram data, average pericardial effusion scores, and histological scores were evaluated by a Student’s *t*-test. The criterion for significance was *p* < 0.05. Data are reported as means ± standard error of the mean (SEM).

## 5. Conclusions

Despite advances in cancer treatment, thoracic cancer survivors treated with RT and receiving cardiac radiation exposure are at risk of complex cardiovascular complications with no known preventative or protective interventions available. Although more studies need to be conducted to elucidate the complex mechanisms of RIHD, recent studies suggest that the immune system may play a role in normal tissue responses to RT, and immune-related adverse effects should be carefully considered when combining RT with immunotherapies. Taken together, the results of this study provide evidence that a full adaptive immune response is not required for rats developing RIHD, and the absence of an intact lymphocyte compartment can lead to differences in radiation-induced cardiac dysfunction. Compared to immune-competent rats, IL2RG^−/−^ rats receiving a high single dose of cardiac RT have poorer heart function measured by echocardiograms than their immune-competent counterparts. This effect was not seen after fractionated RT, with IL2RG^−/−^ rats having slightly less cardiac damage than WT immune-competent rats. Experimental parameters such as animal sex and genetic background, as well as RT dose and fractionation, must be taken into account when determining the effect of adaptive immunity on RIHD. This study provides insights into the considerations needed to evaluate the potential side effects of combinational cancer therapies, such as immunotherapy and RT, and it provides a system to aid in the identification of novel targets for therapeutic interventions that could prevent and/or modify radiation-induced cardiac dysfunction. 

## Supplementary Materials

The following are available online at https://www.mdpi.com/2072-6694/12/4/983/s1, Table S1: Ultrasound parameters for SS WT and IL2RG^−/−^ male and female rats at 3 months after RT, Table S2: Ultrasound parameters for SS WT and IL2RG^−/−^ male and female rats at 5 months after RT.
