# Supplementary material for: Acquired Immunity Is Not Essential for Radiation-Induced Heart Dysfunction but Exerts a Complex Impact on Injury
Source: Cancers (Basel). 2020 Apr 16;12(4):983. doi: 10.3390/cancers12040983 (PMC7226421; doi:10.3390/cancers12040983)
Supplement: Supplementary file 1 [file cancers-12-00983-s001.pdf]

Supplementary Materials

Acquired Immunity Is Not Essential for Radiation-Induced Heart Dysfunction but Exerts a Complex Impact on Injury

Rachel A. Schlaak, Anne Frei, Brian L. Fish, Leanne Harmann, Tracy Gasperetti, Jamie L. Pipke, Yunguang Sun, Hallgeir Rui, Michael J. Flister, Benjamin N. Gantner and Carmen Bergom

Table S1. Ultrasound parameters for SS WT and IL2RG$^{-/-}$ male and female rats at 3 months after RT. Values are means ± SEM; * $p < 0.05$, #$p < 0.01$ for WT versus IL2RG$^{-/-}$.

**Ultrasound Parameters for SS Rats 3 Months Post Radiation**														
**Sex:**	**Female**							**Male**						
**Genotype:**	**WT (*n* = 9)**			**IL2RG$^{-/-}$ (*n* = 14)**				**WT (*n* = 16)**			**IL2RG$^{-/-}$ (*n* = 9)**			
**LVIDd cm/kg**	**2.42**	±	**0.09**	**2.78**	±	0.07	#	1.81	±	0.04	2.06	±	0.08	#
**LVIDs cm/kg**	1.09	±	0.15	1.50	±	0.11	*	0.94	±	0.06	1.23	±	0.06	#
**ESV mL/kg**	0.31	±	0.11	0.52	±	0.10		0.37	±	0.06	0.57	±	0.07	*
**EF %**	87.59	±	4.03	81.59	±	2.67		83.45	±	1.99	76.10	±	2.34	*
**% FS**	56.18	±	5.15	46.73	±	3.02		48.73	±	2.47	40.45	±	2.15	*
**SV mL/kg**	1.72	±	0.11	2.05	±	0.09		1.72	±	0.06	1.81	±	0.10	

Table S2. Ultrasound parameters for SS WT and IL2RG$^{-/-}$ male and female rats at 5 months after RT. Values are means ± SEM; *$p < 0.05$ for WT versus IL2RG$^{-/-}$.

**Ultrasound Parameters for SS Rats 5 Months Post Radiation**													
**Sex:**	**Female**						**Male**						
**Genotype:**	**WT (*n* = 6)**			**IL2RG$^{-/-}$ (*n* = 12)**			**WT (*n* = 14)**			**IL2RG$^{-/-}$ (*n* = 9)**			
**LVIDd cm/kg**	2.68	±	0.11	2.80	±	0.11	1.79	±	0.07	1.96	±	0.09	
**LVIDs cm/kg**	1.33	±	0.14	1.58	±	0.10	0.95	±	0.07	1.18	±	0.05	*
**ESV mL/kg**	0.49	±	0.10	0.58	±	0.11	0.37	±	0.07	0.58	±	0.05	
**EF %**	80.93	±	3.88	78.88	±	2.78	81.92	±	2.67	75.02	±	1.68	
**% FS**	45.87	±	4.41	43.42	±	2.52	46.87	±	2.52	39.22	±	1.44	*
**SV mL/kg**	2.08	±	0.19	2.19	±	0.16	1.63	±	0.15	1.74	±	0.14	

© 2020 by the authors. Licensee MDPI, Basel, Switzerland. This article is an open access article distributed under the terms and conditions of the Creative Commons Attribution (CC BY) license (http://creativecommons.org/licenses/by/4.0/).
